# Prevalence of non-neurological autoantibodies and related comorbidities in stiff person spectrum disorders

**DOI:** 10.3389/fneur.2023.1289460

**Published:** 2023-11-22

**Authors:** Alexandra Balshi, Elena Taylor, Yishang Huang, Danielle Obando, Ashley Miles, Michael Comisac, Yujie Wang, Scott D. Newsome

**Affiliations:** ^1^Department of Neurology, Johns Hopkins University School of Medicine, Baltimore, MD, United States; ^2^Department of Neurology, Beth Israel Deaconess Medical Center, Harvard Medical School, Boston, MA, United States; ^3^Department of Neurology, University of Washington School of Medicine, Seattle, WA, United States

**Keywords:** stiff person syndrome, stiff person spectrum disorders, autoimmune disorders, autoantibodies, disability

## Abstract

**Background:**

Stiff Person Syndrome Spectrum Disorders (SPSD) are a group of rare neurological disorders that can present alongside other autoimmune conditions. However, not much is known about the breadth of non-neurological autoantibodies seen in SPSD nor the observed prevalence of co-existing autoimmune comorbidities and their impact on SPSD.

**Objective:**

This study aimed to investigate the prevalence of non-neurological autoantibodies and associated conditions in a large cohort of people with SPSD.

**Methods:**

A retrospective review of 205 patients with suspected/definitive SPSD seen at Johns Hopkins Hospital from 1997 to 2023 was performed as part of an ongoing, observational study. Relevant demographics, clinical data (e.g., SPSD phenotypes, comorbid conditions, and dates of diagnoses), and laboratory values were collected from electronic medical records. Lab values were excluded if completed within 6 months of receiving intravenous immunoglobin treatment. Summary statistics were performed and assessment for any associations between autoimmune comorbidities and disease burden (modified Rankin score [mRS] and ambulation status) was performed.

**Results:**

The majority of participants had classic SPS (66%), followed by SPS-plus (18%) and PERM (6%) with less than 5% each of the remaining phenotypes and suspected SPS. The average age at symptom onset in this cohort was 44.1 ± 14.5 years (mean ± standard deviation). The majority of the cohort was white (66%) and female patients (75%). The mean mRS was 2.5, and over 70% required assistive devices for ambulation. The most commonly identified non-neurological autoantibodies were anti-nuclear (ANA) (31%), thyroperoxidase (30%), thyroglobulin (20%), and anti-parietal cell (18%) autoantibodies. The most common comorbid autoimmune conditions were autoimmune thyroiditis (38%), insulin-dependent diabetes mellitus (26%), and pernicious anemia (10%). Having more autoimmune comorbidities was weakly associated with higher mRS and a greater need for ambulatory assistance.

**Conclusion:**

The results of this study will hopefully help promote awareness of which autoantibody and medical comorbidity clinicians should be aware of and monitor people with SPSD. Further research is needed to identify if poorly controlled non-neurological autoimmune disorders contribute to disease burden in SPSD and/or if the timing of being diagnosed with one of these conditions plays a role in future disability.

## Introduction

Stiff person syndrome spectrum disorders (SPSD) is an expanding spectrum of autoimmune neurological disorders that most often are associated with autoantibodies against glutamic acid decarboxylase-65 (GAD-65), the rate-limiting enzyme for the synthesis of the inhibitory neurotransmitter gamma-aminobutyric acid (GABA) (1). Less commonly, glycine receptor and amphiphysin antibodies are seen in SPSD. It is unclear to what extent the aforementioned autoantibodies have in the pathogenesis of SPSD; however, it is accepted that these autoantibodies are involved in disrupting the GABAergic pathways, which results in a relatively hyperexcitable nervous system (2). SPSD has several phenotypes that highlight this hyperexcitability—the most common being classic SPS, which presents most commonly as muscle spasms and rigidity of the axial and limb muscles (3).

Individuals with SPSD can have other autoantibodies that are not against neurological antigens and appear to have an increased risk of developing other autoimmune conditions. McKeon and Tracy reported that insulin-dependent diabetes, autoimmune thyroid disease, and pernicious anemia are the most frequent GAD-65 autoimmune associations and that nearly 70% of patients with GAD65 neurological autoimmunity have one or more of these comorbidities (4).

Previous studies also documented the presence of additional serum autoantibodies and their corresponding autoimmune conditions in SPSD (5–9). Dinkel et al. documented islet cell antibodies in 64% (16 of 25), parietal cell antibodies in 28% (7 of 25), thyroglobulin antibodies in 24% (6 of 25), and thyroperoxidase antibodies in 12% (3 of 25) (6). Lee et al. also investigated autoantibody presence in 14 cases of SPSD and reported seropositivity for thyroperoxidase antibodies (2 of 5 tested), ANA (1 of 1 tested), and anti-Ro antibody (1 of 1 tested) (7). Lee et al. also documented cases of comorbid insulin-dependent diabetes mellitus, autoimmune thyroid disease, and Sjögren’s syndrome in 14 SPSD patients.

In our study, we sought to further investigate the breadth of autoantibodies and autoimmune conditions present in the largest cohort of SPSD to date, along with assessing whether those with SPSD and non-neurological autoimmune conditions have a greater disease burden over time.

## Methods

### Patients

This retrospective study was approved by the Institutional Review Board of Johns Hopkins University. All active participants gave written informed consent as part of an ongoing, longitudinal observational study. All participants were seen at Johns Hopkins Hospital between 1997 and 2023 and had a diagnosis of suspected/definitive SPSD. Brain imaging was performed to exclude processes other than SPSD. A total of 205 individuals were identified for inclusion, and only 2% had documented malignancy. Relevant demographics, clinical data such as comorbid conditions and dates of diagnoses, and laboratory investigations were collected via available electronic medical records. SPSD phenotypes were assigned by an expert in these conditions (SDN), and the main descriptors of each phenotype are published elsewhere (10). The patients designated as suspected SPSD had a high clinical suspicion for the condition but did not meet defined criteria (e.g., had low positive serum antiGAD65 antibody or were seronegative). The presence of autoantibodies and comorbidities were assessed across different phenotypes.

### Autoantibodies

Commercially available serum autoantibody laboratory testing was performed as part of routine clinical practice in the care of these individuals. SPSD-associated autoantibody status and type were recorded for each patient. The clinical laboratories for the anti-GAD65 antibody utilized either the enzyme-linked immunoassay (ELISA) method or the radioimmunoassay (RIA) method. For the ELISA method, values at or above 10,000 IU/mL were designated as high, and for the RIA method, the value was at or above 20 nmol/mL. The following autoantibodies were tested: ANA, anti-Beta-2-glycoprotein, cytoplasmic anti-neutrophil cytoplasmic antibody (c-ANCA), perinuclear ANCA (p-ANCA), along with antibodies against cardiolipin, cyclic citrullinated peptide (CCP), double-stranded deoxyribonucleic acid (dsDNA), endomysial, ganglionic acetylcholine receptor, gliadin, La, parietal cell, ribonucleoprotein, rheumatoid factor, Ro, Smith, tissue transglutaminase, thyroglobulin, and thyroperoxidase. Based on previous literature and in our experience, these appear to be the most commonly seen in clinical practice. Other laboratory data are acquired as part of clinical practice (e.g., erythrocyte sedimentation rate) but not included in this study because of the main objectives and purpose of the study.

Each participant was not tested for every autoantibody because some laboratory tests were not available at the time of testing. Patients were considered to have a positive serum autoantibody if they tested positive (based on the resulting laboratory’s value interpretation) for that autoantibody at any point during follow-up. Lab values were excluded if completed within 6 months of receiving intravenous immunoglobin (IVIg) treatment because IVIg can be associated with a falsely elevated autoantibody result (11). To maximize the subjects we could include, we utilized multiple clinically available laboratories and assessed multiple time points.

### Statistical analysis

For patients with any of the three most common autoimmune comorbidities, we recorded their SPSD and comorbidity diagnosis dates. We assessed if comorbidity diagnoses occurred before or after SPSD diagnosis and calculated the mean time between the two diagnoses. Linear regression models were used to assess for any associations between the total number of autoimmune comorbidities and both the modified Rankin Scale (12) ([mRS] 0 = no symptoms;1 = no significant disability; 2 = slight disability; 3 = moderate disability; 4 = moderately severe disability; 5 = severe disability; 6 = dead) and the ambulation status score. An ambulation status score was developed and assigned to each patient using the following criteria: independent as 0; independent except for long distances as 1; cane as 2; bilateral assistance (crutches/walking sticks or walker) as 3; wheelchair as 4; scooter as 5; and bedbound as 6.

## Results

Table 1 outlines the demographic features of the entire cohort (*n* = 205). The average age at symptom onset in this cohort was 44.1 ± 14.5 years. There was a higher female-to-male ratio (~3:1) as seen in other autoimmune disorders, and the majority of the cohort was of white race (~66%). The mean mRS was 2.5, and over 70% required assistive devices for ambulation.

**Table 1:** Overall patients’ characteristics.

Age at symptom onset, years, mean (SD)	44.1 (14.5)
**Time from symptom onset to diagnosis, months, mean (SD)**	53.3 (59.2)
**Female, *n* (%)**	154 (75.1)
**Race, *n*** (%) White Black Asian Other	135 (65.9) 51 (24.9) 5 (2.4) 8 (3.9)
**Ethnicity, *n*** (%) Hispanic	10 (4.9)
**GAD-65 antibody status** Serum positive, *n* (%) Titer IU/mL, mean (SD) CSF positive, *n* (% of tested)	175 (86) 171094 (583353) 52 (59.8)
**Other SPSD antibody status** Glycine serum positive, *n* (% of tested) Amphiphysin serum positive, *n* (%)	11 (5.4) 7 (3.4)
**SPSD phenotype, *n* (%)** Classic SPS SPS-plus Partial SPS Pure CA PERM Possible SPS	135 (66) 36 (18) 8 (4) 6 (3) 12 (6) 8 (4)

SD = standard deviation; GAD-65 = glutamic acid decarboxylase-65; IU/mL, international units/milliliter; SPSD, stiff person spectrum disorder; SPS, stiff person syndrome; CA, cerebellar ataxia; PERM, progressive encephalomyelitis with rigidity and myoclonus.

**Figure 1:**
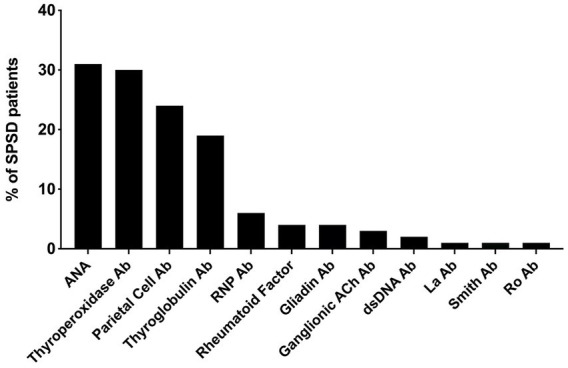
Percent of SPSD patients positive for each non-neurological autoantibody. The most prevalent non-neurological autoantibodies in our SPSD cohort included ANA, thyroperoxidase, parietal cell, and thyroglobulin antibodies. Other autoantibodies were detected in less than 10% of patients. SPSD, stiff person spectrum disorder; ANA, anti-nuclear antibody; Ab, antibody; RNP, ribonucleoprotein; Ach, acetylcholine; dsDNA, double stranded DNA.

**Figure 2:**
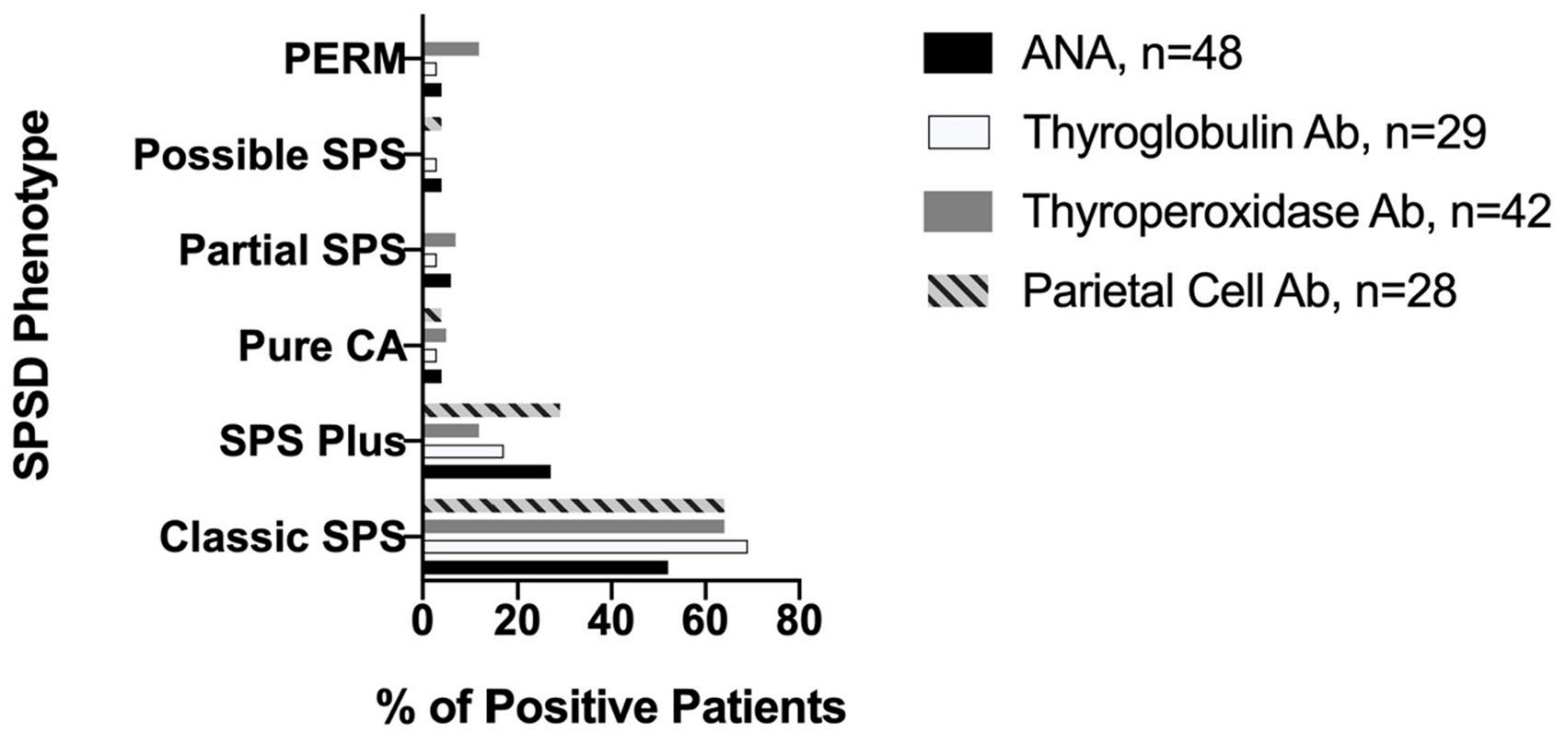
Prevalence of most common non-neurological autoantibodies across SPSD phenotypes. Each of the four most common non-neurological autoantibodies identified in our SPSD cohort (ANA, thyroglobulin, thyroperoxidase, and parietal cell) was found most often in the classic SPS phenotype followed by SPS-plus. SPSD, stiff person spectrum disorder; SPS, stiff person syndrome; PERM, progressive encephalomyelitis with rigidity and myoclonus, CA, cerebellar ataxia; ANA, anti-nuclear antibody; Ab, antibody.

The most commonly identified non-neurological autoantibodies were ANA at 31% (47 of 154), thyroperoxidase at 30% (42 of 138), parietal cell at 24% (28 of 155), and thyroglobulin at 19% (29 of 148). Other non-neurological autoantibodies tested were found in less than 10% of patients. Figure 1 depicts the percentage rates of SPSD patients seropositive for various non-neurological autoantibodies. Individual SPSD phenotypes were also assessed (full details in Figure 2); each of the four most common non-neurological autoantibodies identified were found most often in the classic SPS phenotype followed by SPS-plus.

**Figure 3:**
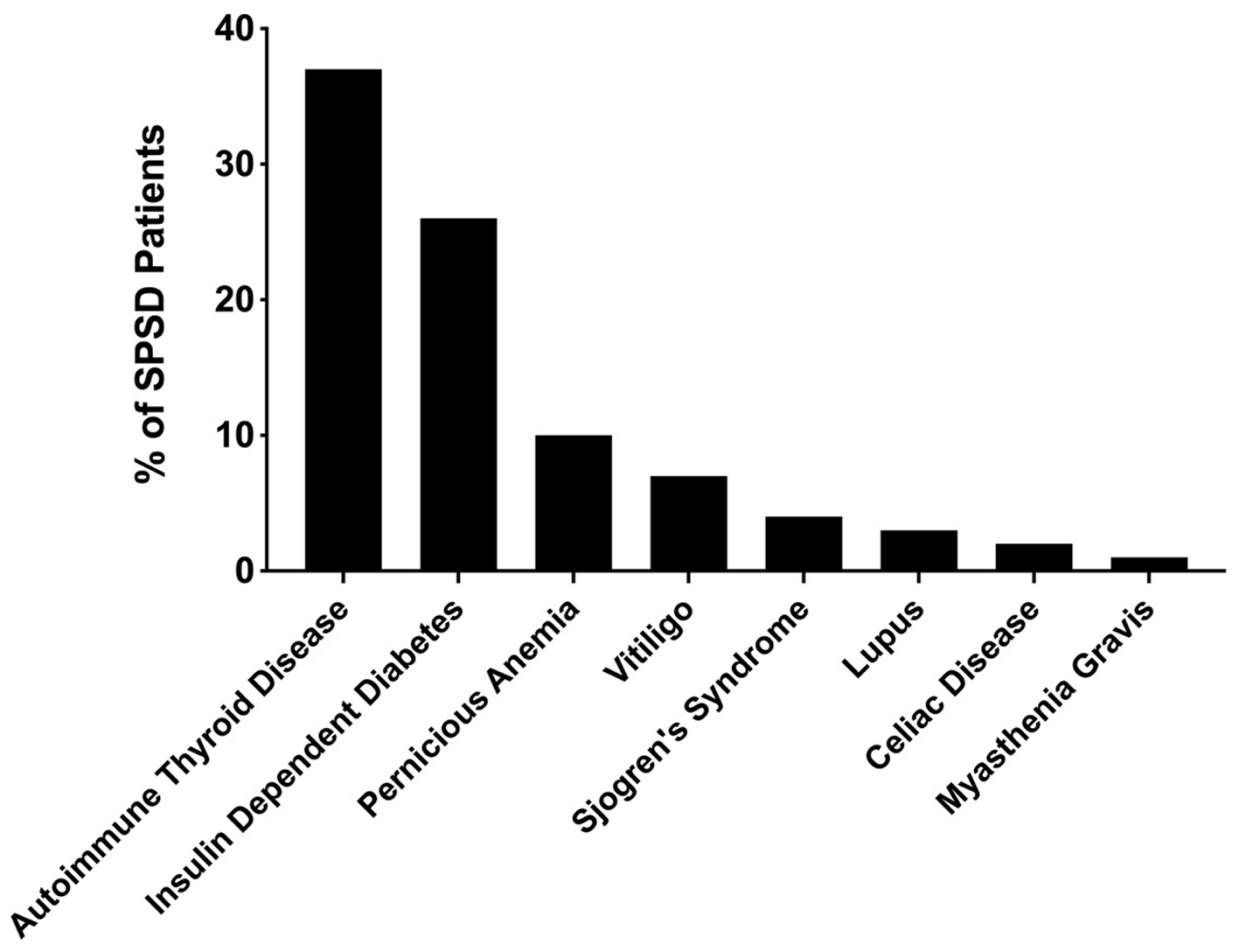
Prevalence of co-existing autoimmune conditions in patients with SPSD. In our SPSD cohort, many patients had multiple autoimmune conditions. Among these, autoimmune thyroiditis was the most prevalent, followed by insulin-dependent diabetes mellitus and pernicious anemia. SPSD, stiff person spectrum disorder.

**Figure 4:**
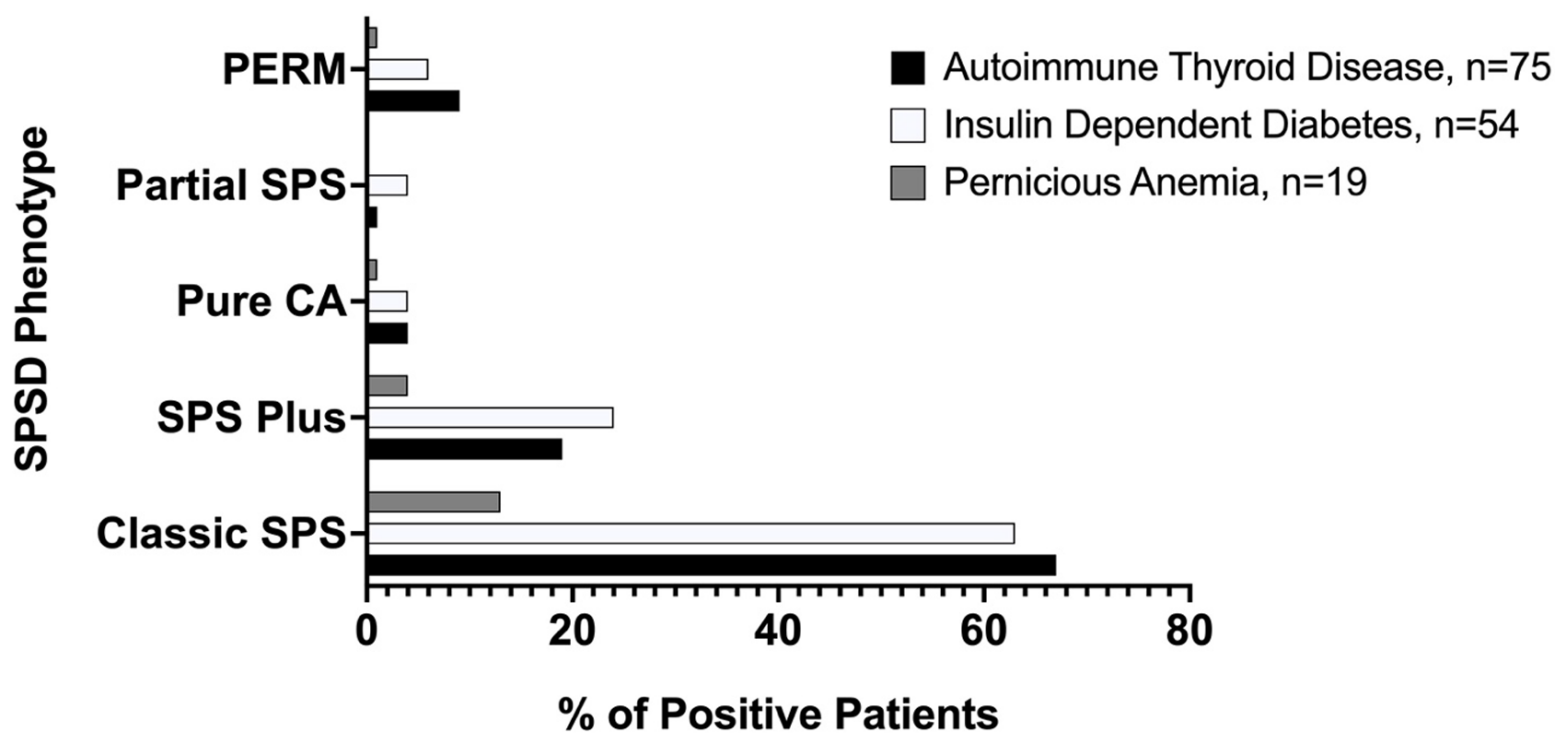
Prevalence of most common medical comorbidities by SPSD phenotype. Autoimmune thyroid disease, insulin-dependent diabetes mellitus, and pernicious anemia were all most common in the classic SPSD phenotype. SPSD, stiff person spectrum disorder; SPS, stiff person syndrome; PERM, progressive encephalomyelitis with rigidity and myoclonus, CA, cerebellar ataxia.

Several co-existing autoimmune conditions were commonly identified in our cohort (Figure 3). The most common of these were autoimmune thyroiditis (38%, 77 of 205) followed by insulin-dependent diabetes mellitus (26%, 54 of 205) and pernicious anemia (10%, 19 of 196). Those with classic SPS had a greater percentage of co-existing autoimmune conditions than other phenotypes especially insulin-dependent diabetes mellitus and autoimmune thyroid disease, as illustrated in Figure 4. In a subgroup analysis that included only those patients with anti-GAD65 antibody seropositivity, the prevalence of non-neuronal autoantibodies and autoimmunity did not change.

For patients with SPSD and autoimmune thyroiditis, the majority (67%) were diagnosed with thyroid disease first at a median of 6.5 ± 9.7 years before SPSD diagnosis. In those diagnosed with thyroid disease after SPSD diagnosis, thyroid disease diagnosis came with a median of 3 ± 3.6 years after SPSD diagnosis. For patients with SPSD and insulin-dependent diabetes mellitus, the majority (63%) were diagnosed with diabetes first at a median of 11.5 ± 10 years before SPSD diagnosis. Those diagnosed with diabetes after SPSD were diagnosed a median of 3 ± 8.5 years later. The majority (50%) of patients with SPSD and pernicious anemia (PA) were diagnosed with PA first at a median of 4 ± 4.3 years before SPSD diagnosis, while those diagnosed with PA after SPSD were diagnosed with PA at a median of 1 ± 1.2 years later.

Linear regression models showed that having more autoimmune comorbidities was weakly associated with greater disability as measured by mRS, with an association of 0.03979 (*p* = 0.49). Therefore, mRS would increase by 0.03979 if the number of comorbidities increased by 1. Comorbid lupus comes with the greatest increase in mRS, with an association of 0.4 (*p* = 0.2). Similarly, the association between a number of autoimmune comorbidities and the ambulatory status score was 0.03624 (*p* = 0.7).

## Discussion

While SPSD is commonly associated with autoantibodies that target the GABAergic pathways, other non-neurological autoantibodies can be present at differing frequencies. However, it remains unclear how common non-neurological autoantibodies occur in SPSD and if there is a predilection of co-existing autoantibodies with a specific SPS phenotype. Additionally, there are limited studies assessing the prevalence of co-existing non-neurological autoimmune disorders (5).

ANA, thyroid autoantibodies (thyroperoxidase and thyroglobulin), and parietal cell autoantibodies were most commonly seen in our cohort. The presence of these autoantibodies helped uncover two of the three most common autoimmune disorders (thyroid disease and pernicious anemia) seen in SPSD. We also found that approximately a quarter of our cohort had insulin-dependent diabetes mellitus. Grimaldi et al., Rakocevic et al., Chia et al., and Budhram et al. found differing percentages of patients in their studies compared to our study patients with autoimmune thyroid disease, insulin-dependent diabetes, and pernicious anemia. These differences could be due to their smaller SPSD cohorts or other factors that are not readily apparent (5, 7, 8, 13).

Nonetheless, the collective literature from larger case series supports that clinicians should be aware of what non-neurological autoantibodies and autoimmune diseases can co-exist with SPSD especially since they can develop after someone is diagnosed with SPSD. Hence, it is important to monitor for the development of these most commonly seen co-occurring autoimmune diseases (e.g., diabetes and thyroid disease). Although the majority of our SPSD cohort had pre-existing comorbidities, many who developed a co-occurring autoimmune comorbidity after SPSD diagnosis did so within a few years. Given this relatively short time, we recommend screening for and monitoring for the development of additional autoimmune conditions so that appropriate referrals and treatments can be initiated.

Our data suggesting a weak correlation in those with SPSD and an increasing number of other autoimmune conditions with disease burden is of interest. Counseling patients on keeping their autoimmune conditions under control is important as a general rule and may be of utmost importance in those with multiple co-existing autoimmune conditions. However, the association seen in our study was small and of unknown clinical significance at this time.

There are some limitations to this study. The laboratory testing for the serum autoantibodies tested was not standardized within one laboratory, and some patients did not have every test completed. Excluding samples within a 6-month IVIg window also narrowed our sample sizes. Additionally, some patients tested positive for an autoantibody at one visit and then negative for the same autoantibody at a later visit. This may have been secondary to other immune therapies that impact the presence of autoantibodies (e.g., rituximab and plasma exchange) although this did not impact the presence of the most common co-existing autoimmune disorders seen in our cohort. Finally, it is not clear if these non-neurological antibodies and/or co-existing autoimmune conditions truly impact disability over time especially if the medical comorbidities are poorly controlled.

Despite these limitations, our study is important as it is the largest study assessing multiple autoantibody seropositivity and autoimmune comorbidities in SPSD. Our results expand our understanding of this rare disorder by documenting the prevalence and type of serum autoantibodies and other autoimmune diseases in SPSD. Furthermore, our data suggest that having these autoimmune comorbidities, especially lupus, may have an impact on disability as what is seen with other autoimmune neurological diseases such as multiple sclerosis (14). However, more research is needed to corroborate such findings and identify if poorly controlled, non-neurological autoimmune disorders contribute to disease burden in SPSD and/or if the timing of being diagnosed with one of these conditions plays a role in future disability.

## Data availability statement

The raw data supporting the conclusions of this article will be made available by the authors, without undue reservation.

## Ethics statement

The studies involving humans were approved by the Johns Hopkins University Institutional Review Board. The studies were conducted in accordance with the local legislation and institutional requirements. The participants provided their written informed consent to participate in this study.

## Author contributions

AB: Conceptualization, Data curation, Formal analysis, Investigation, Methodology, Project administration, Visualization, Writing – original draft, Writing – review & editing. ET: Writing – review & editing, Data curation, Investigation, Visualization. YH: Formal analysis, Writing – review & editing. DO: Writing – review & editing, Data curation, Investigation.AM: Data curation, Writing – review & editing. MC: Data curation, Investigation. YW: Writing – review & editing, Investigation, Supervision, Validation. SN: Conceptualization, Data curation, Investigation, Methodology, Project administration, Resources, Supervision, Validation, Visualization, Writing – original draft, Writing – review & editing.
